# The Power of The (First) Name: Do name tags for operating room staff improve effective communication and patient safety? A proof-of-concept study from an academic medical center in Germany

**DOI:** 10.1186/s13037-024-00418-8

**Published:** 2024-12-09

**Authors:** Alexander D. Bungert, Jan Philipp Ramspott, Carsten Szardenings, Alina Knipping, Benjamin Struecker, Andreas Pascher, Jens Peter Hoelzen

**Affiliations:** 1 Department of General, Visceral and Transplant Surgery, University Hospital Muenster, Muenster, Germany; 2 Institute of Biostatistics and Clinical Research, University of Muenster, Muenster, Germany

**Keywords:** Communication, Risk management, Patient safety, Name tagging, Surgery, Operating room

## Abstract

**Background:**

Effective and reliable communication is the cornerstone of safe communication in the operating room (OR). The OR is one of the most dynamic places in the hospital where multiple disciplines must work together in perfect harmony to ultimately improve patient outcomes. To create familiarity by name regarding constantly changing team members, individual name tagging was implemented in the OR.

**Methods:**

We analysed the impact of name tagging in the OR in a proof-of-concept study. Name tags (either first or last name), coloured according to the specific department, have been placed on the cap since March 13, 2023. On May 26, 2023, a total of 440 anaesthesiologists, general, visceral, and trauma surgeons, nurses, and service staff were invited to answer an evaluation questionnaire of nine questions. The survey period ended on August 7, 2023. 101 people answered the query which, among other things, asked for overall ratings, compliance, evaluation of specific items as well as positive and negative aspects. Statistical analyses were performed using R.

**Results:**

Most of the interviewed staff rated the implementation of name tagging positively (median=3.4; scale from 1-5, 1=bad, 5=good). The greatest benefit was seen in communication in general, direct contact with colleagues, and delegation of tasks. Most of the staff (>90 %) adhered to the new project and used it regularly. Negative aspects mentioned included potential loss of sterility, loss of respectability, and environmental impact. Potential for improvement was seen in the bonding method of attachment or in the implementation.

**Conclusion:**

Individual name tagging in the OR can improve interprofessional communication and is one tool to enhance patient safety by decreasing reservations or intimidations towards previously unknown colleagues. More studies are required to determine long-term effects on patient safety, outcome, or employee satisfaction.

**Supplementary Information:**

The online version contains supplementary material available at 10.1186/s13037-024-00418-8.

## Backround

Ensuring patients' well-being in the operating room (OR) relies heavily on effective communication and strong safety culture [1–3]. Communication is, besides many other definitions, defined as a bidirectional process where information is sent by one party that is unambiguously understood by the receiving party [4]. This transfer is unluckily susceptible to many errors leading to misunderstanding and miscommunication.

However, unequivocal communication between surgeons, anaesthetists, surgical and anaesthetic nurses is essential for high-quality, safe and efficient perioperative patient care. Studies from van Dalen et al. [5] and Birnbach et al. [6] show that people’s names are not known in the OR by the majority despite application of the World Health Organization (WHO) surgical safety checklist (SSC). The poor memorability of names is already known since decades but little has changed since then [7]. Inadequate team communication is still one of the main causes of patient safety impairment in the OR [8, 9]. In the surgical context, up to 56 % of adverse intraoperative and postoperative events are attributable to failures in communication [10, 11] leading to death or loss of function [8].

The “Power of The (First) Name” is a concept that is gaining importance in corporate communications and more and more also in the clinical environment. Addressing employees personally by their (first) name can have a remarkable impact on efficiency and safety in the work environment [12]. Knowing colleagues’ names is a mandatory prerequisite of concepts like the closed loop communication (CLC). Safety culture has its origin in aviation, where Crew Resource Management (CRM) is commonly applied [13]. One tool of CRM is the CLC. This concept is a three-step process where (1) a message is called out to the receiver who is named by its name, (2) the intended receiver accepts the message by repeating the message, and (3) the original sender affirms the correct understanding and interpretation of the message [14]. The CLC concept decreases the likelihood of messages being confused or misunderstood. In a clinical context, such as the OR, sender and receiver of messages are often not in direct eye contact. Here, the CLC concept can make the difference between success and failure, as clear and concise instructions and information are critical.

Status asymmetry between team members may contribute to communication breakdown and threaten patient safety [15, 16]. Addressing employees personally by using their names plays a significant role in minimizing risks and promoting a positive working environment. It creates clear, understandable communication, prevents misunderstandings, helps to reduce hierarchies, and establishes an open culture of dialogue [17]. This concept, the "Power of The (First) Name", deserves our attention in any professional context and especially in clinical practice.

So far, the application of the (first) name in medical context and here especially in the OR has not been largely studied. Therefore, in our study we examined the impact of name tagging in the OR at a university hospital in Germany. Since March 2023, all employees in the operating room have written their name on a sticker put on their forehead. Here, we present the effects of name tagging on employee communication and satisfaction as well as differences between occupational groups.

## Methods

### Study design and hypothesis

In this proof-of-concept study, we retrospectively analysed the impact of name tagging in the OR via questionnaire. We hypothesized that name tags could significantly improve communication in the OR and consequently employees’ satisfaction and patient safety. The project was introduced by the Department of General, Visceral and Transplant Surgery but also conducted by the in-house Department of Trauma Surgery and Department of Anaesthesia of the University Hospital in Muenster, Germany. The roll-out took place across eight shared ORs. The central operating rooms of other departments were excluded due to the pilot character of the study.

### Intervention

**Fig. 1:**
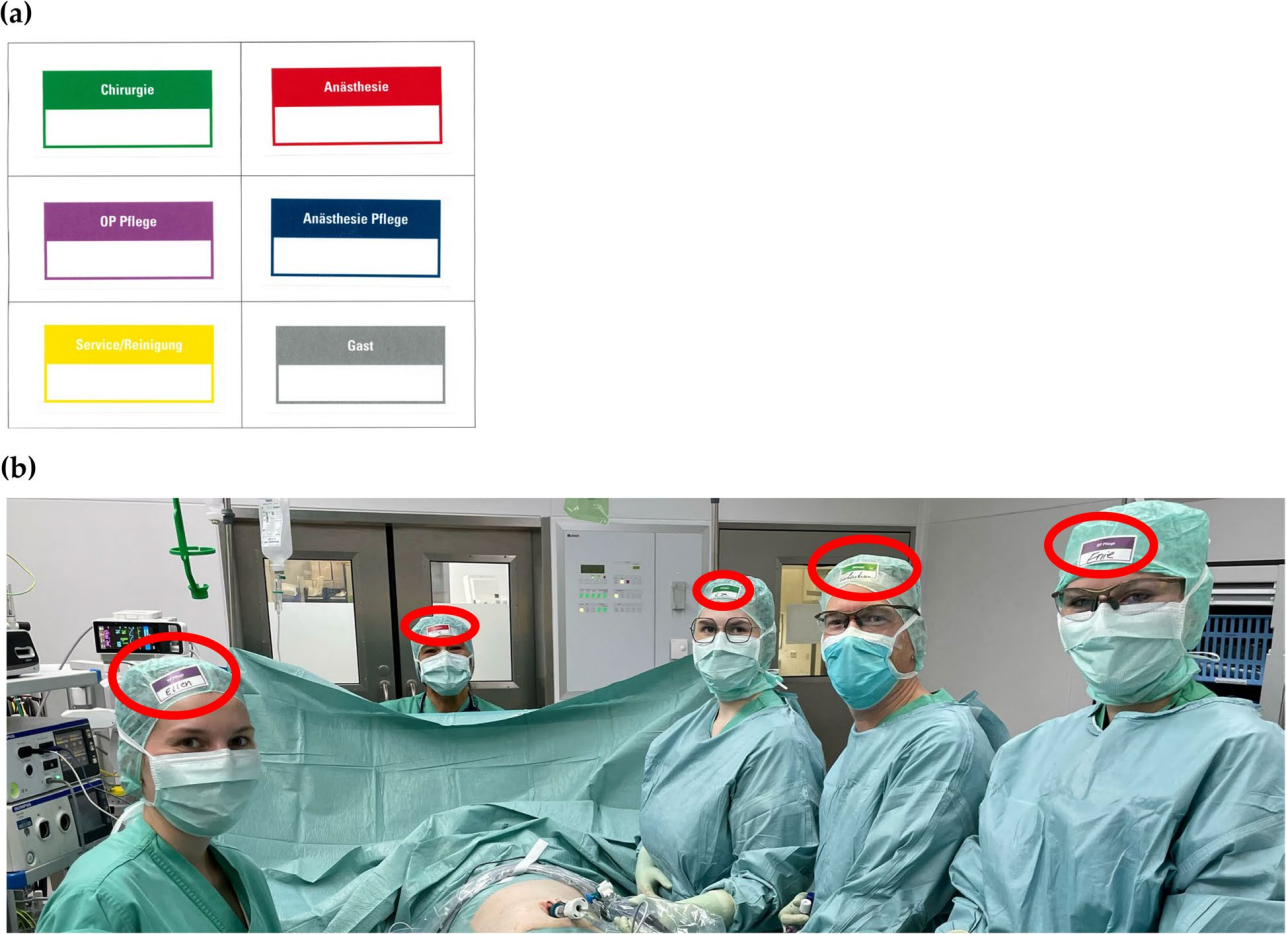
According to the department, name tags were coloured differently and could be filled out individually beneath the profession (white field). Professions were ‘surgery’, ‘anaesthesia’, ‘OR nurse’, ‘anaesthetic nurse’, ‘service/cleaning’ and ‘guest’ (from upper left to bottom right) (**a**). In (**b**) name tags used during surgery are circled. Written informed consent obtained by individuals in the picture is available

Name tags according to personal preference, i.e. first name, last name, both or even nicknames have been put on the bonnet since March 13, 2023. Name tags were also colour-coded and labelled with the professional category ‘surgery’, ‘anaesthesia’, ‘OR nurse’, ‘anaesthetic nurse’, ‘service/cleaning’ or ‘guest’ (Fig. 1).

### Data collection and survey characteristics

On May 26, 2023, a total of 440 anaesthesiologists, general, visceral and trauma surgeons, nurses, cleaning and service staff were invited via email to answer a German-language evaluation questionnaire of nine questions (Supplementary Table S1). Survey period ended on August 7, 2023. Survey was created for this study and evaluated independently by clinical experts to ensure content relevance and validity. Questions were reviewed by clinicians to confirm that questions address all aspects of the intervention’s impact. Reliability was not tested. Main outcome measures were defined retrospectively as overall rating, staff compliance and positive as well as negative aspects. Additional findings could be defined as occupational differences between subgroups.

The questionnaires were handed out with no financial or other non-financial incentives and created by the publicly available online survey tool survio.com (Survio s.r.o., Brno, Czech Republic). The aim of the survey was described in detail in the email and participants’ anonymity and confidentiality were ensured. Duration of the survey was estimated to be five minutes. No time limit to answer the questions was set. Additionally, people were also motivated to forward the email to people not being included in the mailing list. Questions about sex, age, profession, frequency of use, overall rating as well as positive and negative aspects of the project were included. The possibility of free text was given. The free text responses were clustered by two clinical experts, and each cluster was treated as a separate binary variable (mentioned or not mentioned) in the subsequent analysis. Multiple answers were possible.

The study was created with the help of the STROBE cross sectional reporting guidelines [18].

### Statistical analysis

All variables are presented as frequencies and percentages. Accordingly, differences between occupations were tested using Fisher's exact test. Tests were conducted as follows: ‘anaesthetists’ and ‘anaesthetic nurses’ vs. ‘surgeons’ and ’OR nurses’; ‘anaesthetists’ vs. ‘surgeons’; ‘anaesthetic nurses’ vs. ‘OR nurses’ and ‘others’ vs. all other professional groups. All tests were performed with a local significance level of alpha = 0.05. Statistical tests were performed using the program R version 4.3.0 (R Foundation for Statistical Computing, Vienna University of Economics and Business, Vienna, Austria). Due to the exploratory nature of the study no multiple testing correction was applied. All graphs were created using GraphPad Prism version 10.2.3 for Windows, GraphPad Software, Boston, Massachusetts USA, www.graphpad.com.

## Results

### Study population

This study involved 101 people who completed the questionnaire. Employees’ sociodemographic characteristics are presented in detail in Table 1. Most of the participants were between 31 and 40 years old (*n*=45, 44.6 %). Gender was equally distributed between male (*n*=49, 48.5 %) and female (*n*=50, 49.5 %) participants. Respondents were equally distributed between the surgical department (surgeons (*n*=29; 28.7 %) and OR nurses (*n*=17; 16.8 %)) and anaesthetic department (anaesthetists (*n*=29; 28.7 %) and anaesthetic nurses (*n*=20; 19.8 %)) with 45.5 % and 48.5 %, respectively. Further professional groups (e.g. service and cleaning staff) were summarized under ‘others’.

**Table 1:** Sociodemographic data of all study participants

	**N**	**%**
**Age (years)**		
21–30	21	20.8
31–40	45	44.6
41–50	22	21.8
51–60	10	9.9
> 60	3	3.0
**Sex**		
male	49	48.5
female	50	49.5
unspecified	2	2.0
**Profession**		
surgeons	29	28.7
OR nurses	17	16.8
anaesthetists	29	28.7
anaesthetic nurses	20	19.8
others	6	5.9

### Overall rating and frequency of use of name tagging in the OR

**Fig. 2:**
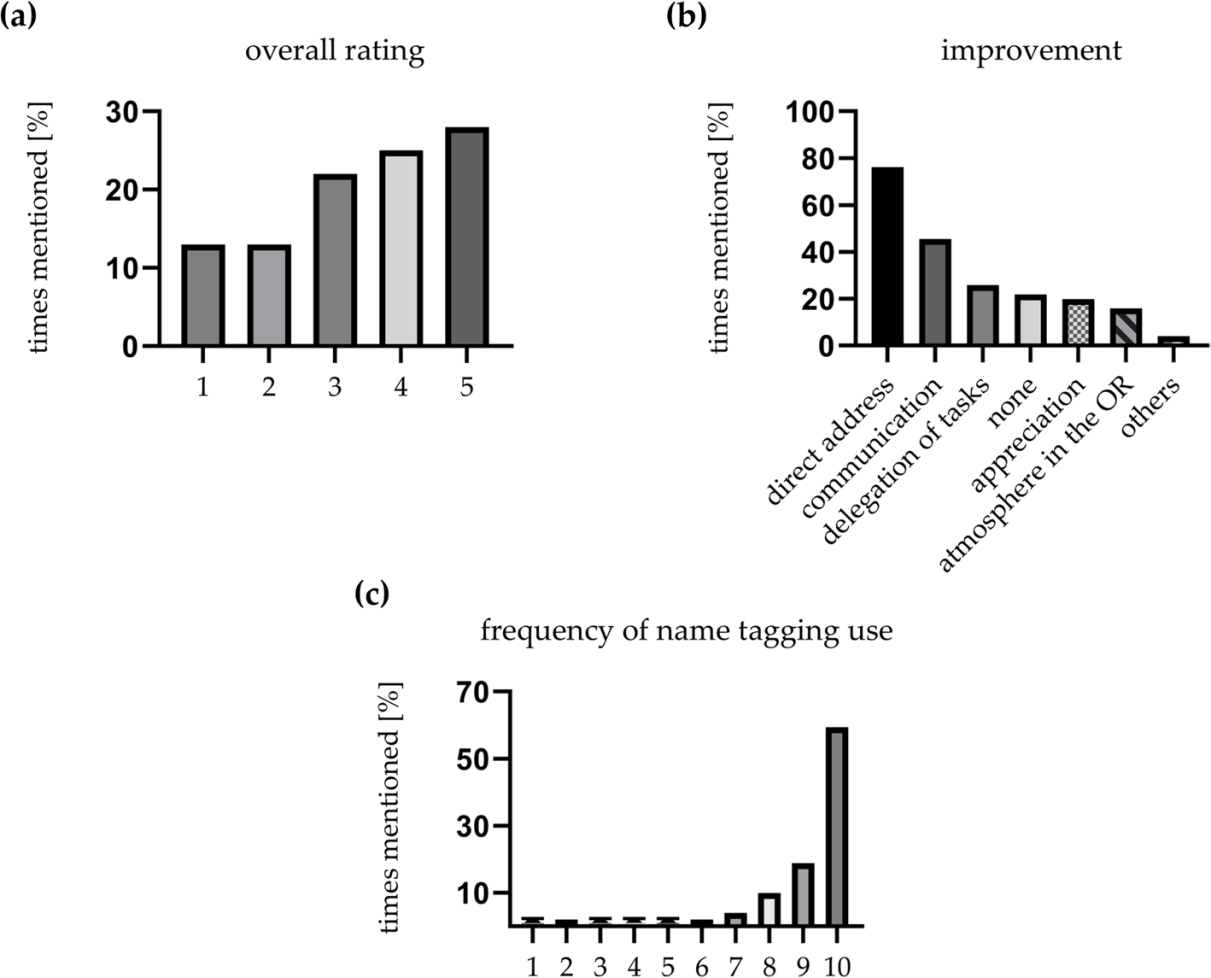
Overall rating, aspects of improvement and frequency of use of name tagging in the OR. The total score (**a**) shows a general evaluation of the project (range from 1 = bad to 5 = good, median = 3.4). Positive effects are shown in (**b**). The frequency of use is illustrated in (**c**) (range from 1 = never to 10 = always). Overall rating was very good (**a**), improvement was mostly seen in direct address through using names and communication in general (**b**). Overall compliance was high (**c**)

The overall assessment of the project was mostly positively rated and the majority of the respondents rated at least 3 (74.23 %) (range from 1 to 5, 1: bad, 5: good). The median rating was 3.42 (Fig. 2a). Most of the participants (76.2 %) selected the direct address in the OR as a positive aspect of improvement. It is followed by enhanced communication in general with 45.5 % nominations. The third most frequently selected facet is delegation of tasks (25.7 %). Remarkably, for 21.8 % of the employees nothing has changed. Personal appreciation and the atmosphere in the OR have only changed for 19.8 % and 15.8 %, respectively (Fig. 2b). The overall frequency of participation was very high. Most of the people used name tagging regularly: 18.8 % and 59.4 % rated 9 and 10, respectively (Fig. 2c, range from 1 – 10, 1: never, 10: always).

### Negative aspects and potential improvement of name tagging in the OR

**Fig. 3:**
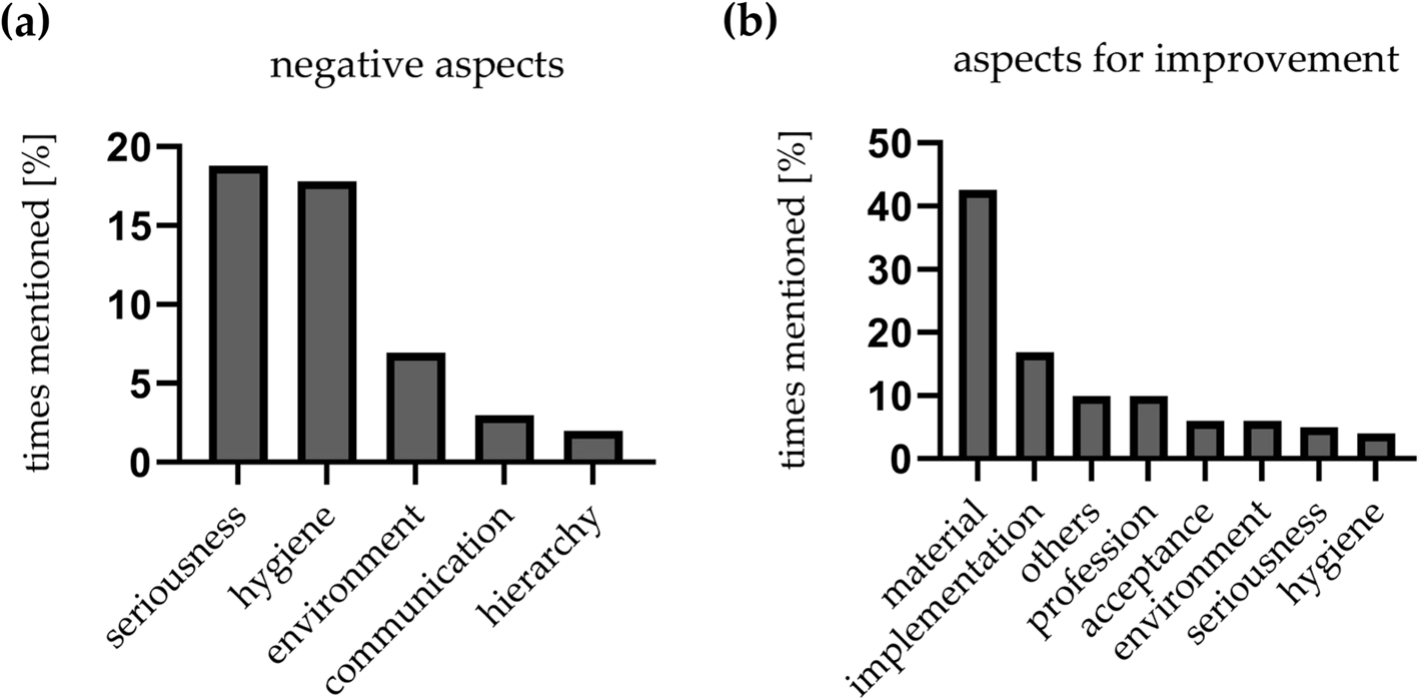
Negative aspects and aspects for improvement of use of name tagging in the OR. The mentioned negative (**a**) as well as the potential aspects for improvement (**b**) were given in free text and clustered according to the figures. Multiple answers were possible. Seriousness and hygiene were amongst the most mentioned negative aspects (**a**) whereas the material of the name tags and the implementation were most in need of improvement (**b**)

In the questionnaire, we explicitly asked about negative aspects of name tagging in the OR. Given answers were clustered in five groups. Most nominations could be attributed to hygienic aspects and seriousness (17.8 % and 18.8 %). Lack of hygiene was associated with poor adhesive function of the sticker and thus the potential risk of falling into the operating site. Seriousness was attributed to the adhesion site of the sticker itself. Sticking something on his head was considered as kind of humiliation. For some people, the use of stickers represents an environmental pollution (6.9 %). Impairment of communication and hierarchy in the OR was only seen as negative for 3 % and 2 % of the participants, respectively (Fig. 3a). The questionary also asked for further suggestions to ameliorate the project. Accordingly, negative aspects like little adhesion power of the stickers was considered as being one main factor to improve and was therefore mentioned by 42.6 % of all respondents and was also subsumed under ‘hygiene’ (4 %). The same applies to the previously mentioned site of application of the stickers – it is reflected in the implementary aspect (16.8 %). The reusability of the name tags was important for 5.9 % of the employees (‘environment’). Under ‘others’ things like redundancy are pointed out. The reason given is that by using the already existing Surgical Safety Checklist (SSC) where all persons present in the OR should introduce themselves at the beginning of the surgical intervention, another tool for mutual introduction would be obsolete. Moreover, communication between different specialties should be improved in general through communication trainings (‘others’, 9.9 %). Another point that was mentioned is that not all persons in the OR are well reflected by the predetermined groups on the name tags. For example, trainees or interns of the nursing school do not find a corresponding designation on the name tags (9.9 %). For 5.9 % of the participants the acceptance of the project played a dominant role. For example, it has been noticed that within the participating departments not all staff members were convinced and even made fun of name tagging (‘acceptance’). The danger of losing respectability was seen by 5 % (‘seriousness’). There is a fear that patients might mistake the stickers on the head of the anaesthetic staff as a game and consequently this would not express the seriousness of the situation before a surgical intervention (Fig. 3b).

### Occupational-specific factors of improvement and impairment

#### Specific factors of improvement

**Fig. 4:**
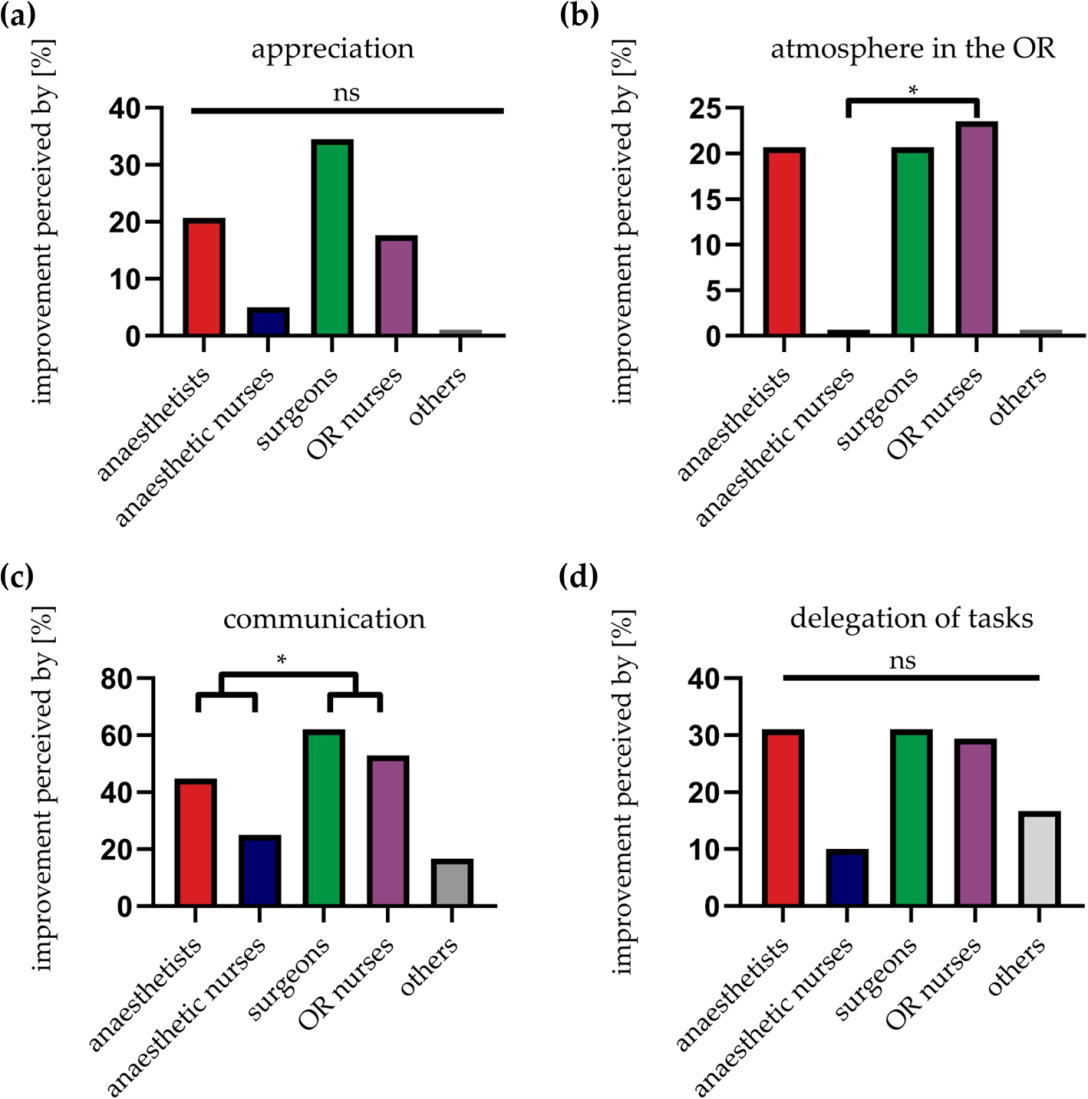
Occupational specific factors of improvement after implementation of name tagging in the OR. Columns are illustrated in accordance with the occupational specific colour of the name tags (except for ‘others’). Appreciation (**a**) did not change significantly between the professional groups. Although, the atmosphere in the OR was enhanced for ‘OR nurses’ compared to ‘anaesthetic nurses’ (**b**). Communication shows significant amelioration on part of the surgical department (**c**) whereas the delegation of tasks does not seem to differ between professional fields (**d**). ns = not significant, *P < 0.05; Fisher's exact test

In Fig. 2b we presented the results regarding positive changes after implementation of the name tagging project in general. We were wondering if results could differ between the different specialties as the contact with the patient is diverse. Therefore, we asked for occupational-specific factors of improvement and impairment of name tagging in the OR. Concerning the improvement of ‘appreciation’, there was no statistically significant difference between the subgroups ‘anaesthetists’, ‘anaesthetic nurses’, ‘surgeons’, ‘OR nurses’ and ‘others’. However, there is a striking variance in the number of nominations between ‘surgeons’ and ‘anaesthetic nurses’ (34.5 % vs. 5 %). Other occupational groups (‘others’) did not cast a vote (Fig. 4a). The atmosphere in the OR is considered as being enhanced by mainly the surgical department and even significantly different between ‘OR nurses’ and ‘anaesthetic nurses’ (23.5 % vs. 0 %) with no votes by ‘anaesthetic nurses’ and ‘others’ (Fig. 4b). Communication was found to be ameliorated by rather surgical subgroups and least for ‘others’ (62.1 % for ‘surgeons’ and 52.9 % for ‘OR nurses’ vs. 44.8 % for ‘anaesthetists’ and 25 % for ‘anaesthetic nurses’, 16.7 % for ‘others’) (Fig. 4c). However, again 16.7 % of this professional category found the delegation of tasks to be enhanced. Between all professional groups no statistically significant difference was observed although the surgical department did cast more votes (‘surgeons’ 32.8 % and ‘OR nurses’ 23.5 % vs. ‘anaesthetists’ 25.9 % and ‘anaesthetic nurses’ 7.5 %) (Fig. 4d).

#### Specific factors of impairment

**Fig. 5:**
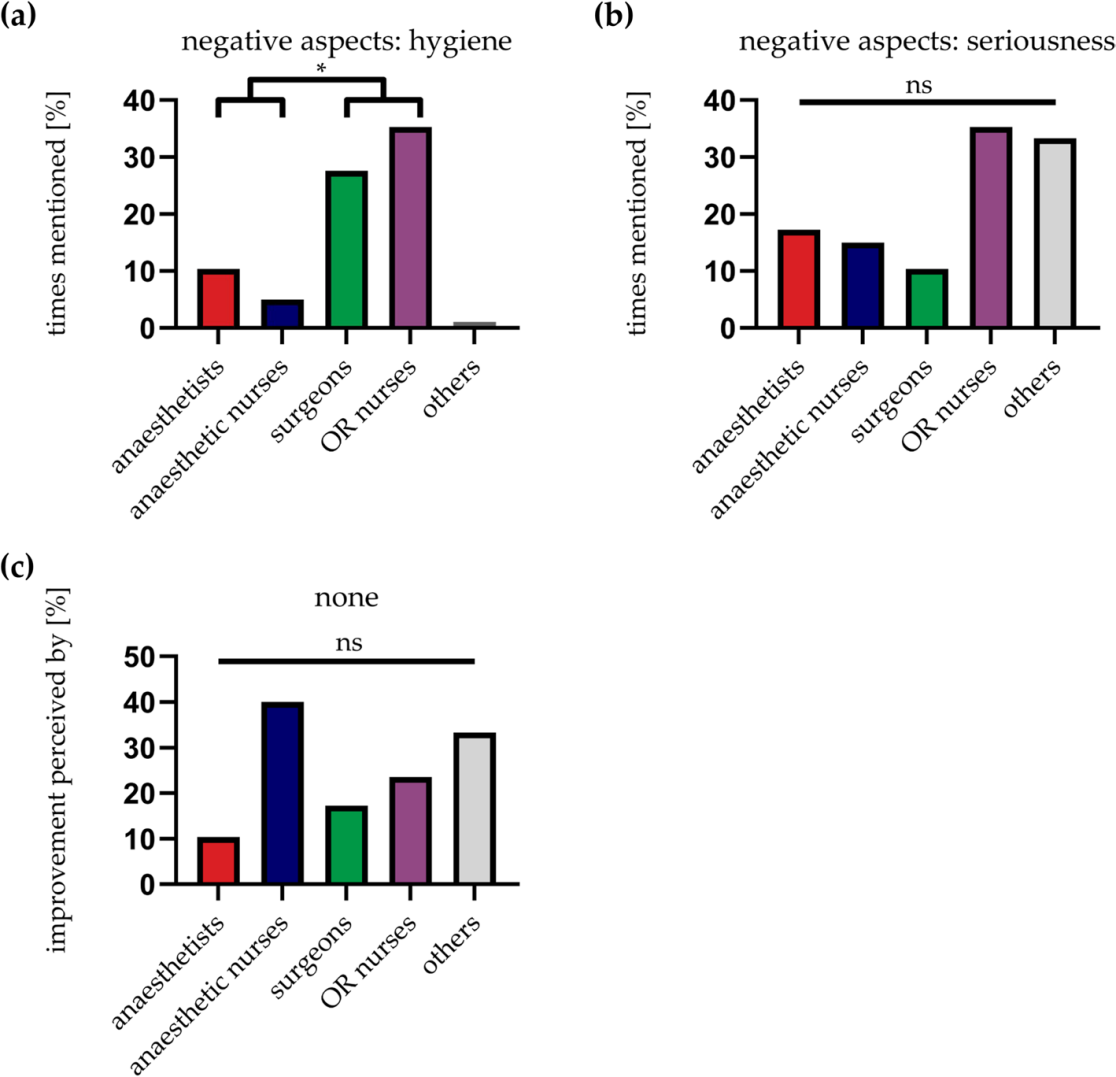
Occupational specific negative aspects after implementation of name tagging in the OR. Columns are illustrated in accordance with the occupational specific colour of the name tags (except for ‘others’). The lack of hygiene was significantly mentioned more often by ‘surgeons’ and ‘OR nurses’ compared to ‘anaesthetists’ and ‘anaesthetic nurses’ (**a**). Seriousness is most likely to be seen as impaired by ‘OR nurses’ and ‘others’ (**b**). No improvement was most frequently seen among participants in anaesthesia care (**c**). ns = not significant, *P < 0.05; Fisher's exact test

In our study we also asked for occupational specific negative aspects after introducing the name tagging. The negative hygienic aspect was significantly more important for ‘surgeons’ and ‘OR nurses’ compared to ‘anaesthetists’ and ‘anaesthetic nurses’ (27.6 % for ‘surgeons’ and 35.3 % for ‘OR nurses’ vs. 10.3 % for ‘anaesthetists’ and 5 % for ‘anaesthetic nurses’) (Fig. 5a). Impairment of the external representation (‘seriousness’) was most likely considered critically by ‘OR nurses’ and ‘others’, whereas there was no significant difference to the other professional groups (35.3 % for ‘OR nurses’ and 33.3 % for ‘others’) (Fig. 5b). Numerically, the introduction of name tagging in the OR has changed the least for anaesthesia care and ‘others’ compared to the other professional fields (‘anaesthetic nurses’ 40 % and ‘others’ 33.3 % vs. 10.3 %, 17.2 % and 23.5 % for ‘anaesthetists’, ‘surgeons’ and ‘OR nurses’) (Fig. 5c).

## Discussion

### Main findings and current literature

Here we present the results of newly introduced name tagging in the OR and its perception. Most of the interviewed staff showed good compliance and rated the implementation of name tagging positively with greatest benefit in communication. It is noteworthy that satisfaction and agreement of anaesthesia care is the lowest amongst all professions. We could only speculate that this is most likely since attendance in the OR is also at its lowest due to numerous other and sometimes parallel tasks that need to be completed. In the study by Vats et al. implementation of the SSC was tested in 2010 [19]. Here, routine use was limited when staff felt embarrassed by formal introduction by name or use was not considered good by the supervisor. This could also apply for our name tags as names are visible for everyone in the OR and rejection of the project is at least conceivable in rare cases. Besides, material and seriousness were criticized the most. The name tags used in our study were relatively cost-effective in this pilot trial and are planned to be replaced by stickers with enhanced adhesive strength. According to the free texts of the questionnaire the aspect of seriousness is based most likely on the forehead position of the name tags. Colleagues recognized the fear being considered dubious by patients although patient assessment was not done and therefore remains unclear. In principle, name tagging serves to minimize risks in the OR and increase patient safety. Communication risks generally arise from the fact that recipients are not named or only named unspecifically by calling out the profession for example, thus preventing a two-way communication. On the other hand, working in an OR where colleagues are known by name and profession leads to a more familiar environment where things can be addressed at a low threshold. We could show that name tagging could also lead to better perception as a team member and therefore to better execution of tasks [20]. Moreover, patients can identify the physicians which are currently taking care of them creating a better doctor-patient relationship [21]. The idea of name tags has become famous by introducing the TheatreCapChallenge in the United Kingdom [22] creating a prerequisite for a clear two-way communication. This has become even more important since introduction of robotically assisted surgery that goes hand in hand with spatial separation and increasing noise levels [23]. During robotically assisted surgery, the surgeon sits at the console without direct eye contact to his colleagues. The spoken word is transmitted to the operating table via microphone and loudspeaker. The surgeon is therefore dependent on his commands being understood, as otherwise the patient may be injured if instruments are moved or exchanged without prior consultation. In this case, poor communication leads to poor patient outcome [23]. Communication in the OR is closely linked to job satisfaction [24]. Moreover, shortage of skilled labour in Europe is worrying and leads to more and more temporary labour. This applies even more to university hospitals with a high number of employees that often do not know each other. Heterogeneity of surgical and non-surgical teams rises and harbours incalculable risks. Therefore, methods of risk minimization are urgently required. Through the introduction of the SSC and instruments of CRM communication should be improved and standardized. Nevertheless, our study has shown the advantages of individual name tagging as well as potential aspects of improvement. Generally, name tagging is one tool to enhance interprofessional communication and consequently patient safety with de facto no drawbacks.

### Limitations and strengths

First, our study is a non-validated study as we did not conduct a formal pilot test. A formal test-retest reliability assessment was not performed due to time constraints and the explorative nature of the study. Another limitation of our study is the relatively low response rate of our questionnaire with 23 %. This could lead to biased results as non-participating people could eventually reject the idea of name tagging and therefore weaken the significance of our (mostly) positive results. Unsurprisingly, participation is particularly low among the service and cleaning staff (summarized under ‘others’). This may be related to the fact that German is not their native language and direct communication between service and cleaning staff and other professional groups is generally less frequent and harbours little potential for conflict. Participation itself may also be low because no time limit was set for answering the questionnaire. Another limitation is that we did not conduct a follow-up to determine long-term effects and if use is sustained. One major strength of our study is that survey was distributed via email without major hurdles. Moreover, questions were few, easy to understand and intervention as well as aim of the study were straightforward and clear.

## Conclusion

Even though using name tags in the OR is already known it is still not widely applied. By now, there is a lack of research how and which communication name tags could exactly improve in the OR. This study underlines that name tagging in the OR can significantly improve communication by, among other things, dismantling hierarchies and better execution of tasks leading to enhanced teamwork, which may positively impact patient safety. The study highlights a need for further investigation into long-term effects on patient outcomes and staff satisfaction, especially as communication challenges grow with the use of robotic surgeries. Name tagging is seen as a promising tool and could prompt policymakers to standardize name tagging in hospitals. Improvements in design and implementation are needed to create acceptance in all professional groups and to reduce reservations.

## Supplementary Information

Supplementary Material 1: Table S1: Questionnaire

## Data Availability

No datasets were generated or analysed during the current study.

## Abbreviations

OR: Operating room
WHO: World Health Organization
SSC: Surgical Safety Checklist
CLC: Closed Loop Communication
CRM: Crew Resource Management
