# Supplementary material for: The Power of The (First) Name: Do name tags for operating room staff improve effective communication and patient safety? A proof-of-concept study from an academic medical center in Germany
Source: Patient Saf Surg. 2024 Dec 9;18:35. doi: 10.1186/s13037-024-00418-8 (PMC11629488; doi:10.1186/s13037-024-00418-8)
Supplement: Supplementary file 1 — Supplementary Material 1: Table S1: Questionnaire [file 13037_2024_418_MOESM1_ESM.docx]

Supplementary Material Table S1: Questionnaire

| What things have positively changed with the introduction of name tagging? | Direct address |
| --- | --- |
|  | Communication |
|  | Appreciation |
|  | Delegation of tasks |
|  | Atmosphere in the OT |
|  | None |
|  | Others (free text) |
|  | |
| What things have negatively changed with the introduction of name tagging? | Free text |
|  | |
| Would you like to see this concept transferred to the central OT? | Yes |
|  | No |
|  |  |
| How do you rate this project? (from 1=bad to 5= good) | 1-5 |
|  | |
| How often have you used the name tag in the OT? (from 1= never to 10= always) | 1-10 |
|  | |
| Further aspects for improvement | Free Text |
|  | |
| Which professional group do you belong to? | Surgery |
|  | Anaesthesia |
|  | OT nurse |
|  | Anaesthetic nurse |
|  | Trainees |
|  | Students |
|  | Service staff |
|  | Cleaning staff |
|  | Others (free text) |
|  | |
| How old are you? | 21-30 |
|  | 31-40 |
|  | 41-50 |
|  | 51-60 |
|  | >60 |
|  | |
| Which gender do you feel do you belong to? | Male |
|  | Female |
|  | Diverse |
